# Fabrication of a Hierarchical Bioinspired Super-Contrast Wettability Surface for Enhanced Atmospheric Water Collection

**DOI:** 10.3390/biomimetics11100759

**Published:** 2026-10-20

**Authors:** Ming Li, Bo Wang, Bingyang Wang, Yanling Wan

**Affiliations:** 1 School of Intelligent Manufacturing and Electrical Engineering, Guangzhou Institute of Science and Technology, Guangzhou 510540, China; lmingdr@163.com; 2 College of Mechanical and Automotive Engineering, Ningbo University of Technology, Ningbo 315211, China; bywang2024@nbut.edu.cn; 3 Ministry of Education Key Laboratory of Cross-Scale Micro and Nano Manufacturing, Changchun University of Science and Technology, Changchun 130022, China; wanly@cust.edu.cn

**Keywords:** super-contrast wettability, nanosecond laser, Ti-6Al-4V, atmospheric water collection, Laplace pressure

## Abstract

With a growing population and severe water pollution, worldwide water scarcity has become a serious problem. In this context, collecting water from the atmosphere is regarded as a promising route to alleviate the global water shortage. Inspired by indocalamus leaves, desert beetle backs and cactus spines, we report a hierarchical bioinspired super-contrast wettability surface (BSCWS) fabricated on Ti-6Al-4V alloy via nanosecond laser selective processing and chemical modification. The BSCWS exhibits extreme contrast wettability with superhydrophobic regions (water contact angle: 167.4 ± 1.1°) and superhydrophilic regions (water contact angle: 0°). The mechanism underlying the extreme wettability transition was elucidated through systematic chemical composition analysis. Furthermore, a novel type of three-level BSCWS was designed, which can realize large-area water collection, directional transport and centralized storage. Under 100% relative humidity, the three-level BSCWS achieves a collection efficiency of 203.1 ± 10.8 g/m^2^·h, which is 1.3, 3.3, 5.6 and 8.0 times higher than those of the two-level BSCWS, superhydrophilic, superhydrophobic and smooth surfaces. A dual Laplace pressure-driven mechanism is proposed to explain the enhanced atmospheric water collection performance. This work provides a scalable and light-weight strategy for high-efficiency atmospheric water collection.

## 1. Introduction

Water scarcity, exacerbated by rapid global population growth and severe water pollution, has emerged as a critical challenge constraining sustainable human development. Against this background, atmospheric water collection is widely regarded as a promising and sustainable strategy to alleviate water crises in arid regions due to its ubiquitous availability [1,2,3,4,5]. However, achieving high-efficiency atmospheric water collection remains a significant challenge. Over millions of years, nature has developed complicated surface wettability regulation strategies that offer profound inspiration for water collection solutions [6,7]. For instance, the Namib desert beetle utilizes a hydrophilic/hydrophobic alternating microstructure on its back to capture and transport fog droplets directionally [8]; cactus spines utilize the Laplace pressure difference induced by their gradient curvature to drive spontaneous liquid transport from tip to base [9]; spider silk employs periodic spindle-knot structures for efficient water collection [10]. Nevertheless, water collected by a single bioinspired strategy is often dispersive and difficult to transport promptly, which leads to evaporation losses, reduced efficiency and delayed collection processes [11]. Consequently, integrating multiple biomimetic models to construct patterned bioinspired superwetting surfaces has emerged as a key strategy to potentially improve water collection efficiency [12]. However, current fabrication strategies are predominantly centered on constructing patterned surfaces through chemical etching [13,14,15], chemical vapor deposition [16,17], template methods [18,19], etc. These methods are often plagued by complex processing procedures, expensive costs and difficulties in achieving large-area fabrication, which limit their practical application.

In recent years, laser processing technology has emerged as an effective method to construct biomimetic functional micro/nanostructures because of its non-contact nature, high precision and exceptional versatility [20]. Specifically, a nanosecond laser is particularly well suited for large-scale industrial production for its superior processing efficiency and significantly lower equipment costs compared with femtosecond and picosecond lasers [21,22]. Consequently, using laser processing technology to combine surface functions from multiple biomimetic models and build patterned bioinspired superwetting surfaces has become a promising strategy for scalable, cost-effective and highly efficient water collection. For example, Hou et al. developed superhydrophilic/superhydrophobic hybrid (SSH) surfaces for enhanced water collection by fabricating superhydrophilic triangular-patterned arrays on a superhydrophobic copper substrate via laser processing [23]. Similarly, Liu et al. [24] constructed a superhydrophobic/superhydrophilic wedge-shaped patterned surface (SSWPS) and investigated the influence of microstructures on water collection performance. Both studies mentioned above, inspired by the desert beetle backs and cactus spines, constructed wedge-shaped arrays of superhydrophilic regions on superhydrophobic substrates to achieve rapid water directional transport and superior collection efficiency. Based on this, introducing leaf-vein-inspired or tree-shaped hierarchical structures can further enhance water transport and collection performance [15,25,26,27]. Wang et al. constructed a bioinspired hybrid surface featuring superhydrophilic vein-like patterns on superhydrophobic (SHB) copper by chemical etching, 3D printing and laser scanning. This design successfully realized a highly efficient and stable water collection property [15]. Lin et al. developed leaf-vein-inspired hierarchical wedge-shaped superhydrophilic regions on a flexible PET substrate via a low-temperature and alkali-assisted surface oxidation process to grow Cu(OH)_2_ nanowires [27]. The optimized four-level structure achieved water collection efficiency approximately 11.5 times greater than that of a bare PET substrate. Wang et al. developed a highly efficient water collection system by fabricating tree-shaped hierarchical cones on a superhydrophobic TiO_2_ film via laser direct writing, achieving a collection efficiency approximately 36 times greater than that of the superhydrophobic surface [25].

Regardless of whether constructing superhydrophilic wedge arrays or hierarchical vein-like or tree-shaped patterns on superhydrophobic substrates, the underlying mechanism is essentially the same: the process of collection is achieved through Laplace pressure differences or surface energy gradients [15,24]. Although these designs enable rapid directional water transport, they are inherently limited in that the water is only collected locally and dispersed on the surface, and gravity is required to drive the water to roll off the surface. Even when gravity facilitates the removal of part of the condensed droplets, a large amount of water remains pinned on the superhydrophobic substrate, which significantly reduces the overall water collection efficiency. In addition, most existing wedge or vein-like patterned surfaces primarily collect water from narrow hydrophilic regions. Large-area water collection on the surface cannot be achieved by simply enlarging the hydrophilic area. Consequently, it is highly desirable to develop new hierarchical patterns, which can achieve a large-area and full-coverage water-collection strategy.

Moreover, we note that research on metallic substrates has predominantly focused on aluminum and its alloys [24,28], stainless steel [29,30] and copper and its alloys [15,23]. Investigations involving Ti-6Al-4V remain comparatively scarce. Although Wang et al. and Liu et al. recently explored the influence of superhydrophobic surface adhesion on water collection performance on Ti-6Al-4V [31,32], the construction of super-contrast wettability surfaces on this alloy has rarely been reported. The present study selects Ti-6Al-4V alloy as the substrate material for two main reasons. First, Ti-6Al-4V possesses excellent corrosion resistance and outstanding environmental stability under high-humidity atmospheric environments [22,33,34], which can effectively prolong the service life of collection devices; second, Ti-6Al-4V exhibits an outstanding specific strength (strength-to-density ratio) [31,33]. Although its mass density (~4.4 g cm^−3^) is higher than that of aluminum alloys (~2.8 g cm^−3^), its superior specific strength enables the fabrication of thinner-walled device structures. This characteristic provides significant light-weighting potential for portable and miniaturized outdoor water collection equipment. Therefore, adopting Ti-6Al-4V alloy as the substrate offers promising application prospects and practical research value for atmospheric water collection.

In this study, based on the well-known contrast wettability of desert beetle backs and the directional liquid self-transport of cactus spines, we herein introduce a novel biomimetic strategy: the anisotropic wettability of indocalamus leaves. The lower surface of indocalamus leaves exhibits excellent anisotropic wettability [31], which can enable directional droplet movement along the parallel veins while hindering movement in the perpendicular direction. Inspired by these biomimetic principles, we reported a novel three-level BSCWS fabricated on Ti-6Al-4V alloy via nanosecond laser selective processing and chemical modification, which can realize large-area capture, directional transport and centralized storage of collected water. The extreme wettability contrast and its transition mechanism were systematically investigated through surface characterization and chemical composition analysis. The water collection performance of the three-level BSCWS was evaluated and compared with surfaces of different levels and wettability. In addition, a dual Laplace pressure-driven mechanism was proposed to elucidate the whole collection process of the three-level BSCWS. This work aims to provide a simple and scalable strategy for large-area and high-efficiency atmospheric water collection with significant light-weighting potential for practical applications.

## 2. Materials and Methods

### 2.1. Materials

Ti-6Al-4V alloy plates (20 mm × 20 mm × 2 mm) were purchased from Huijing Metal Materials Co., Ltd. (Dongguan, China), and its chemical composition consisted of 5.5–6.75% Al, 3.5–4.5% V and balance Ti. Prior to laser processing, the samples were polished using SiC sandpapers with #1000, #1500 and #2000 grits sequentially for 5 min each to remove surface oxides and machining scratches. Subsequentialy, the substrates were ultrasonically cleaned in acetone (40 mL), ethanol (40 mL) and deionized water (40 mL) (Changchun Hongda Fine Chemical Co., Ltd., Changchun, China) for 10 min each to ensure a contaminant-free surface. To decrease the surface energy, 1H, 1H, 2H, 2H-perfluorodecyltrimethoxysilane (FAS-17, 97%, Shanghai Macklin Biochemical Technology Co., Ltd., Shanghai, China) was dissolved in ethanol with a mass fraction of 1 wt%, which served as the chemical modification agent [31,35]. All reagents were of analytical grade and used as received without further purification.

### 2.2. Methods

**Figure 1:**
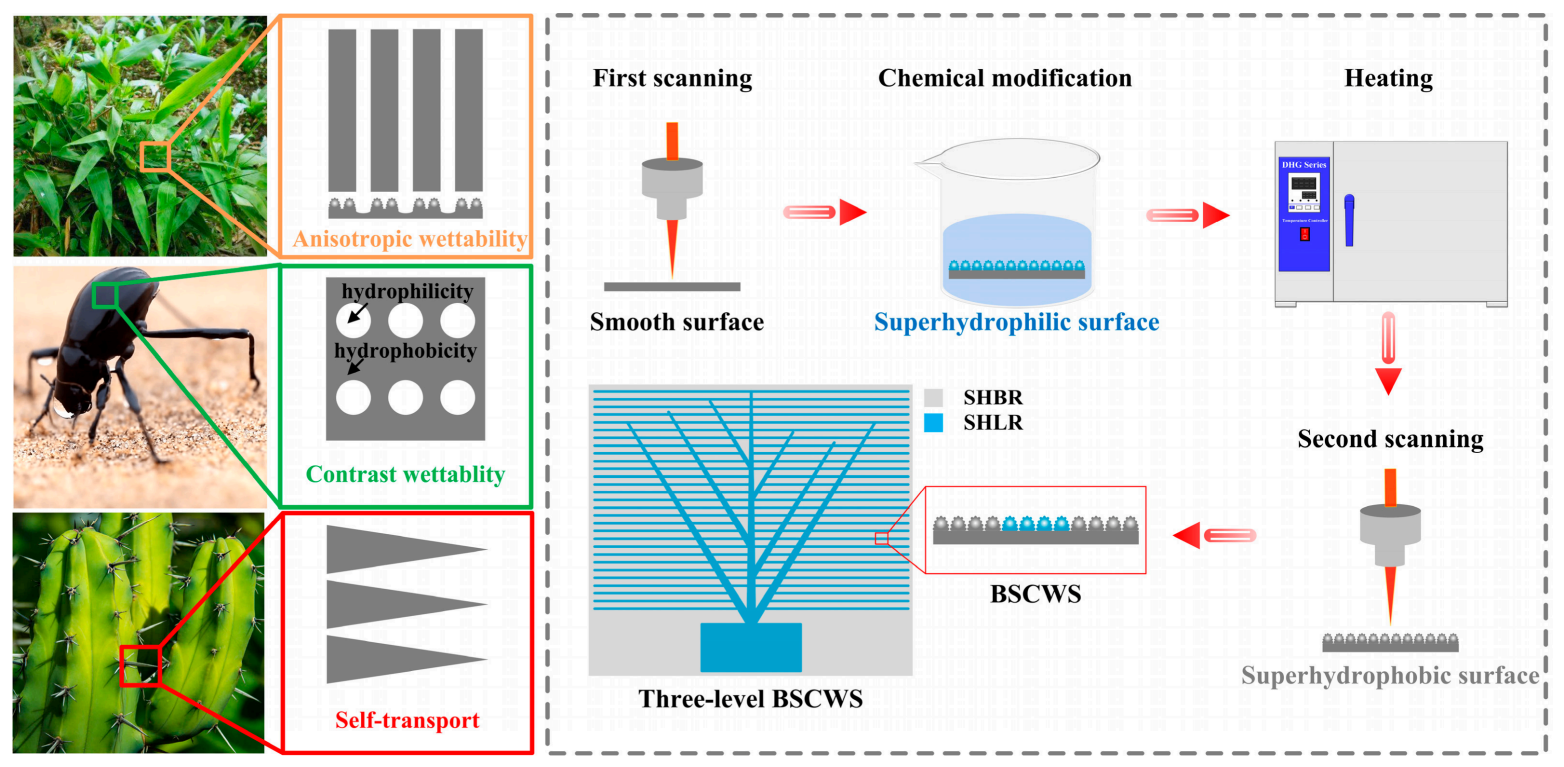
Schematic diagram of the bioinspired design and laser fabrication process for the BSCWS.

A nanosecond laser system (FB20-SBGZ, Changchun New Industries Optoelectronics Tech. Co., Ltd., Changchun, China) was used to fabricate the hierarchical micro-/nanostructures on Ti-6Al-4V alloy, and the detailed processing parameters are listed in Table 1. The fabrication process of the BSCWS involved a three-step strategy, as illustrated in Figure 1. The polished, smooth surface was processed by first laser scanning to create the superhydrophilic surface. Then the laser-processed surface was immersed in the 1 wt% chemical modification agent for 2 h and subsequently cured in a drying oven at 100 °C for 1 h to obtain a uniform superhydrophobic surface. Finally, a second laser scanning was performed on the superhydrophobic surface to fabricate the BSCWS, which featured coexisting superhydrophobic (SHBR) and superhydrophilic (SHLR) regions.

### 2.3. Characterization

Surface morphologies were characterized using a scanning electron microscope (SEM, ZEISS EVO 18, Oberkochen, Germany) and a confocal laser scanning microscope (CLSM, Olympus OLS5100, Tokyo, Japan). The surface chemical composition was investigated via energy-dispersive X-ray spectroscopy (EDS, JEOL JED-2300, Tokyo, Japan) and X-ray photoelectron spectroscopy (XPS, Thermo Fisher Scientific Escalab 250Xi, Waltham, MA, USA). Surface wettability was evaluated by measuring the water contact angle (WCA) and water sliding angle (WSA) using an optical contact angle measurement system (OCAMS, KRüSS DSA 25S, Hamburg, Germany). To ensure statistical reliability, each measurement was repeated five times under ambient temperature (~25 °C). The process of water collection was recorded by the industrial digital microscope (DM201, Shenzhen Yongan Technology Co., Ltd., Shenzhen, China).

## 3. Results and Discussion

### 3.1. Morphologies of the BSCWS

**Figure 2:**
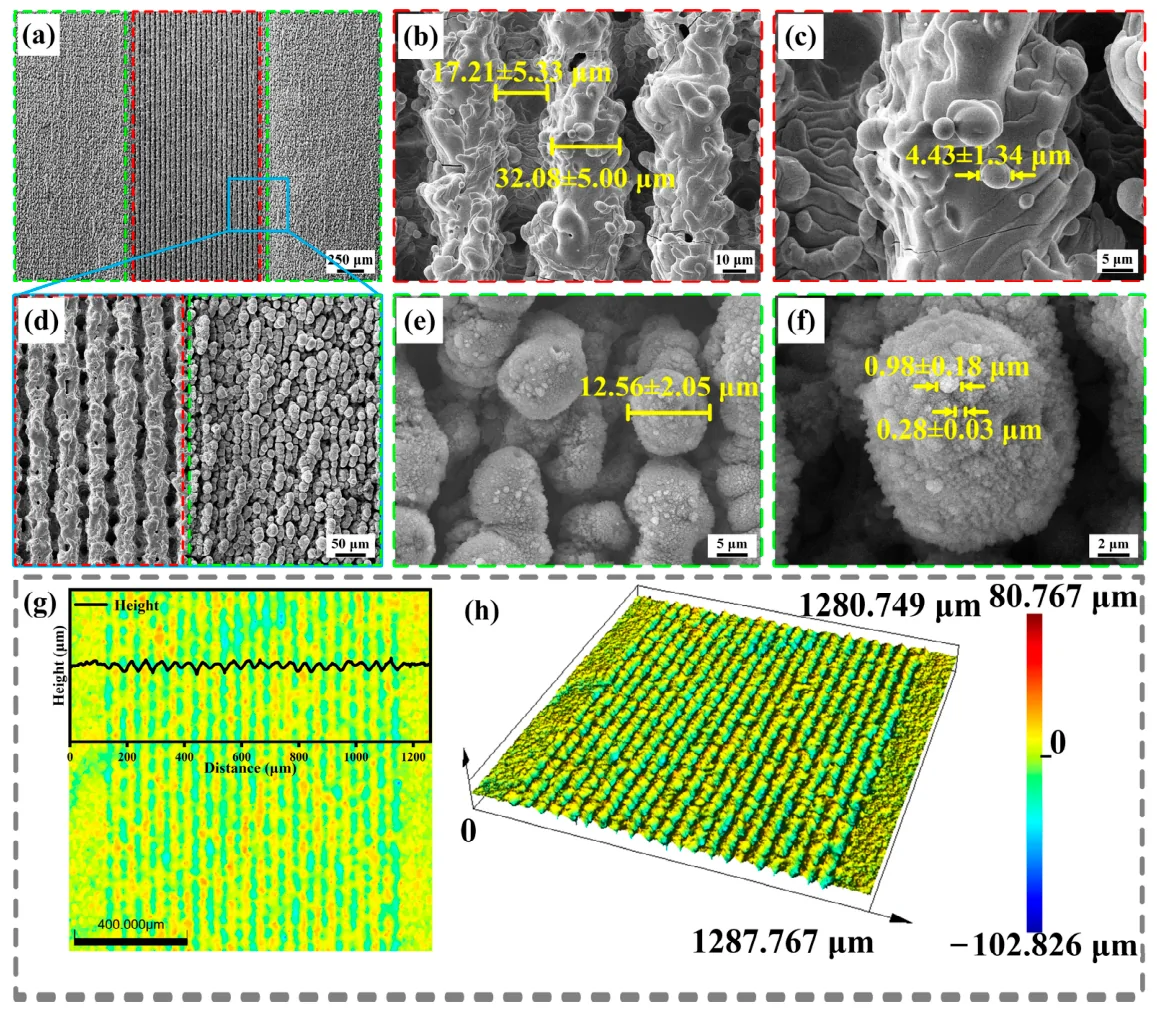
Morphologies of the BSCWS: (**a**,**d**) SEM images of the BSCWS; (**b**,**c**) high-magnification SEM images of the SHLR; (**e**,**f**) high-magnification SEM images of the SHBR; (**g**,**h**) 3D morphologies and cross-sectional profile of the BSCWS.

Figure 2 illustrated the morphologies of the fabricated BSCWS. The BSCWS exhibited distinct patterned boundaries including an SHBR (green dashed boxes) and SHLR (red dashed boxes), as shown in Figure 2a,d. The SHBR featured densely populated micro-scale protrusions covered with numerous nanoscale particles (Figure 2e,f). The micro-scale protrusions on the SHBR had an average size of 12.56 ± 2.05 μm. The nanoscale particles formed submicron clustered structures with different sizes, in which the larger clusters measured 0.98 ± 0.18 μm and the smaller ones were 0.28 ± 0.03 μm. Such micro/nanostructures were generated by severe laser–material interaction [31]. During laser processing, the laser scanning interval (3 μm) was much smaller than the laser spot diameter (50 μm), resulting in substantial overlap between adjacent scanning lines. The structures processed previously underwent repeated ablation during subsequent laser scanning. Driven by the cumulative effect of cyclic ablation and solidification during repeated laser processing, the hierarchical micro/nanostructures were ultimately formed.

In contrast, the SHLR displayed periodically arranged micro-scale grooves (Figure 2b,c). This structure arose from the larger scanning interval (50 μm) used during the second laser scanning, which weakened the interaction between adjacent laser scanning paths. The formation of the ridge-like structure was attributed to the accumulation and solidification of the ejected melt. The micro-scale groove width was 17.21 ± 5.33 μm, and the micro-scale ridge structure between neighboring grooves possessed an average width of 32.08 ± 5.00 μm. In addition, numerous spherical solidified droplets were observed adhering to these ridge surfaces (Figure 2c), which had an average diameter of 4.43 ± 1.34 μm.

Figure 2g,h present the 3D morphologies of the BSCWS. A sharp contrast was observed between the SHBR and SHLR: the SHLR exhibited distinct wave-like undulations in its cross-sectional profile, while the flanking SHBR remains relatively flat (Figure 2g). To quantitatively analyze the surface morphologies, five key surface roughness values of BSCWS, SHBR and SHLR, including Sa (arithmetical mean height), Sz (maximum height), Sq (root mean square height), Sp (maximum peak height) and Sv (maximum valley depth), were measured by CLSM, as shown in Table 2. The SHLR exhibited the highest roughness, with an Sa of 9.55 ± 0.29 μm and Sz of 139.67 ± 9.11 μm. The BSCWS showed intermediate roughness values (Sa = 8.50 ± 0.15 μm and Sz = 139.43 ± 9.91 μm). The SHBR possessed the lowest Sa of 5.38 ± 0.38 μm and Sz of 64.40 ± 12.52 μm. Additionally, Sq, Sp and Sv followed an identical trend: SHLR > BSCWS > SHBR. In summary, the surface morphology and roughness were essential for achieving the distinct wettability of both regions. Although SHBR possessed lower values for all roughness values (Sa, Sz, Sp, Sv and Sq) compared with the SHLR, sufficient hierarchical micro-scale protrusions with nanoparticles rather than high roughness were the key factor for realizing superhydrophobicity [36,37]. These typical structures provided abundant room to trap air to stabilize the Cassie–Baxter state for superhydrophobicity [38]. In contrast, the high roughness of the SHLR originated from large-scale open micro-scale grooves fabricated by the second laser scanning. The high roughness amplified the surface wettability and enhanced the capillary action, driving the surface to a superhydrophilic state [31].

### 3.2. Chemical Compositions of the BSCWS

**Figure 3:**
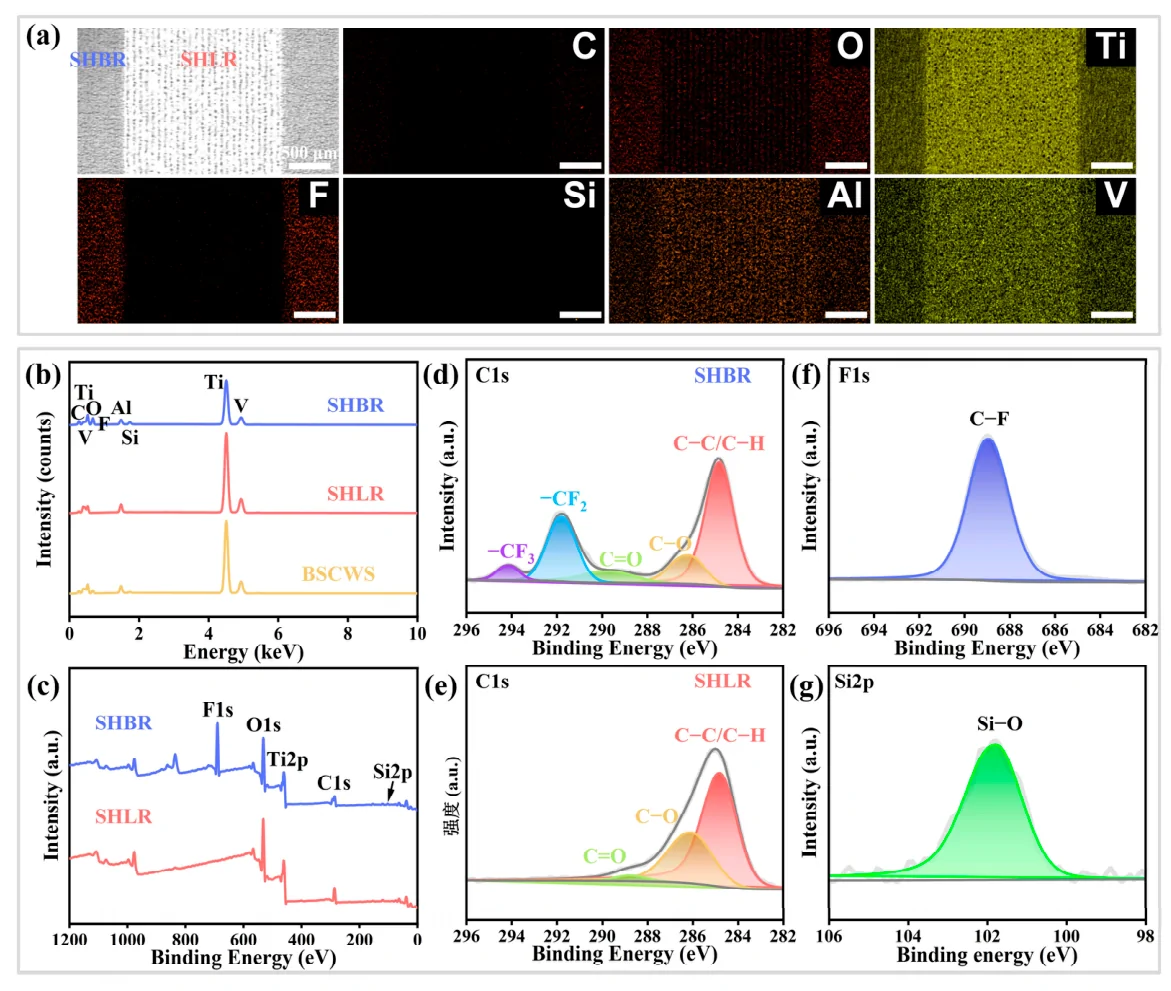
Chemical composition of the BSCWS, SHBR and SHLR: (**a**) elemental mapping of the BSCWS; (**b**) EDS spectra of the BSCWS, SHLR and SHBR; (**c**) XPS survey spectra of the SHBR and SHLR; (**d**,**e**) high-resolution C1s spectra of the SHBR and SHLR; (**f**,**g**) high-resolution F1s and Si2p spectra of the SHBR.

The extreme wettability contrast was governed by the synergistic effect of the surface morphology and chemical composition [39,40]. To elucidate the transition of the wettability, the chemical composition of the BSCWS, SHBR and SHLR were analyzed using EDS and XPS (Figure 3). As shown in Figure 3a, the elemental mapping of the BSCWS revealed a uniform distribution of the O, Ti, Al and V elements. Notably, the C and F elements were predominantly localized in the SHBR, whereas Si was present in trace amounts with an indistinct distribution. The EDS spectra of the BSCWS, SHLR and SHBR are presented in Figure 3b. Both the BSCWS and SHBR possessed seven elements (C, O, Ti, Al, V, F and Si), whereas the SHLR consisted of only five elements (C, O, Ti, Al, and V). This indicated the complete depletion of F and Si on the SHLR following the second laser processing. As summarized in Table 3, the C content decreased from 10.61% to 4.10%, F dropped from 22.58% to 0%, and O increased from 43.16% to 50.47%. These results preliminarily suggested that the extreme wettability transition from superhydrophobicity to superhydrophilicity was driven by the depletion of C and F, coupled with the increase in O.

The deep mechanism of the extreme wettability transition was not elucidated precisely through elemental content variations, XPS was further employed to analyze the chemical composition of the SHLR and SHBR. As shown in the XPS survey spectra (Figure 3c), the SHBR exhibited additional F1s and Si2p peaks compared with the SHLR. The corresponding high-resolution spectra (Figure 3f,g) confirmed the presence of F and Si in the SHBR, which was consistent with the EDS results (Figure 3b). The high-resolution C1s spectra of the SHBR and SHLR are displayed in Figure 3d,e. The C1s spectrum of the SHBR can be deconvoluted into five peaks at binding energies of 284.80, 286.15, 289.61, 291.78 and 294.11 eV, corresponding to the C−C/C−H, C−O, C=O, −CF_2_ and −CF_3_ groups, respectively [41,42]. The −CF_2_ and −CF_3_ groups originated from fluorosilane (FAS-17) molecules. In contrast, the C1s spectrum of the SHLR was deconvoluted into three peaks at 284.80, 286.07 and 288.75 eV, corresponding to the C−C/C−H, C−O and C=O groups, respectively. The complete disappearance of the −CF_2_ and −CF_3_ groups in the SHLR after second laser processing was primarily attributed to the thermal decomposition of fluorosilane molecules. Specifically, the decomposition of the −CF_2_ and −CF_3_ groups initiated above 250 °C and was nearly complete at 450 °C [43,44]. Given that the melting temperature of the Ti-6Al-4V alloy during laser processing was approximately 1600 °C, it far exceeded the complete decomposition temperature of the fluorosilane molecules. Furthermore, as indicated in Table 4, the oxygen-containing-group content in the SHLR was 35.98%, which was 1.75 times higher than that in the SHBR (20.60%). Therefore, the superhydrophobicity of the SHBR was primarily contributed by the fluorinated groups, while the extreme transition from superhydrophobicity to superhydrophilicity was induced by the thermal decomposition of these fluorinated groups and the concurrent increase in oxygen-containing groups [41].

### 3.3. Wettability and Analysis of the BSCWS

**Figure 4:**
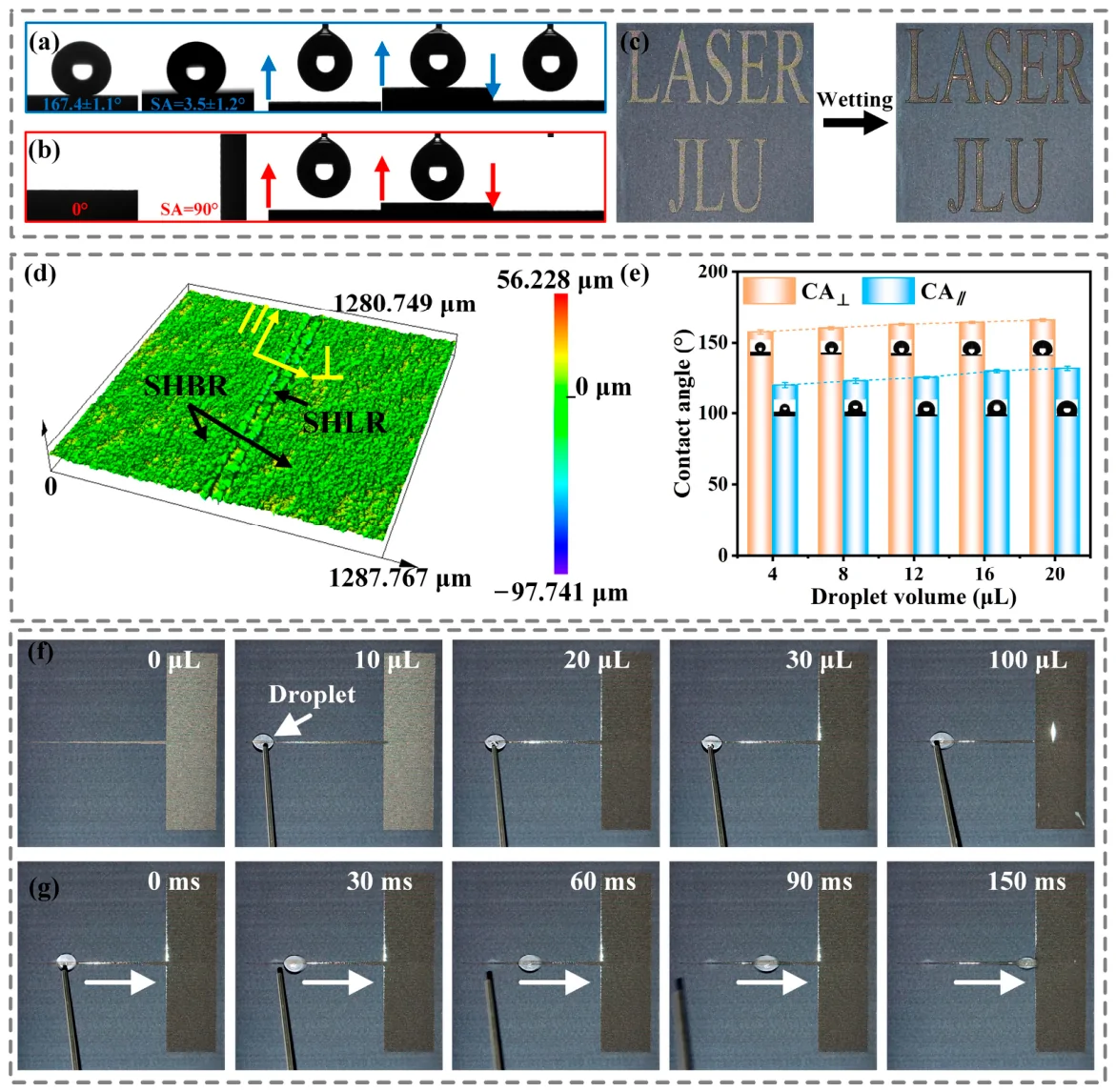
Wettability of the BSCWS, SHLR and SHBR: (**a**,**b**) wettability of the SHBR and SHLR; (**c**) special-patterned BSCWS of English texts; (**d**) CLSM of the BSCWS with rectangular SHLR; (**e**) anisotropic wettability of the BSCWS with rectangular SHLR; (**f**,**g**) water directional transport and storage process of the BSCWS with wedge-shaped and rectangular SHLRs.

Figure 4 illustrates the wettability of the BSCWS, SHBR and SHLR. As shown in Figure 4a,b, the SHBR demonstrated extreme superhydrophobicity with a WCA of 167.4 ± 1.1° and WSA of 3.5 ± 1.2° (Figure 4a), while the SHLR possessed a WCA of 0° and WSA of 90° (Figure 4b), showing excellent superhydrophilicity. Specifically, the water droplet cannot adhere to the SHBR (Figure 4a) due to the superhydrophobicity and low adhesion force (~15.7 μN, as shown in Figure S1a). Conversely, upon contact with the SHLR with extreme superhydrophilicity, the droplet immediately spread across the surface to form a continuous water film (Figure 4b). The BSCWS with superhydrophilicity and superhydrophobicity can achieve precise wettability control by adjusting the SHLR. The wettability of the BSCWS featuring an SHBR with a rectangular SHLR (Figure 4d) is shown in Figure 4e. The parallel and perpendicular directions were defined as *∥* and ⊥ in Figure 4d. As the droplet volume increased from 4 μL to 20 μL, WCAs in the perpendicular direction were 157.0°, 160.5°, 162.6°, 163.7° and 165.2°, whereas WCAs in the parallel direction were 120.2°, 122.2°, 125.6°, 129.3° and 130.7°, which exhibited pronounced WCA anisotropy. In addition, the WSAs in the perpendicular direction were always larger than those in the parallel direction (Figure S1b). By analyzing the WCAs and WSAs, we demonstrated that the surface exhibited excellent anisotropic wettability. This anisotropic wettability occurs due to the substantial interfacial energy barrier existing in the wettability boundary between the SHBR and SHLR [43,45,46]. When the droplet contacts the SHLR, it is captured by strong interfacial adhesion and tends to spread along the parallel direction. In contrast, droplet movement in the perpendicular direction to the SHLR channel is strongly restricted because of the high interfacial energy barrier. Such anisotropic behavior guides liquid to propagate preferentially along superhydrophilic channels for continuous liquid collection. Based on this theory, we designed a BSCWS for the spontaneous directional collection of water by adjusting the geometry of the SHLR. Figure 4f presents a self-driven droplet transport track and storage reservoir composed of wedge-shaped and rectangular SHLRs. A 10 μL droplet was continuously dispensed at the apex of the wedge-shaped SHLR and continuously flowed into the rectangular reservoir. The detailed process is shown in Figure 4g, the droplet was released at 0 ms and reached the storage reservoir within 150 ms. Furthermore, to demonstrate the versatility of this design, we customized the SHLR into English texts (“LASER” and “JLU”). As shown in Figure 4c, these specific SHLRs were fabricated onto the SHBR background. Upon contact, droplets selectively entered these SHLRs and were strictly confined by the wettability boundaries, which successfully formed novel super-contrast wetting patterns.

### 3.4. Water Collection Performance of the Three-Level BSCWS

#### 3.4.1. Design of the Three-Level BSCWS

**Figure 5:**
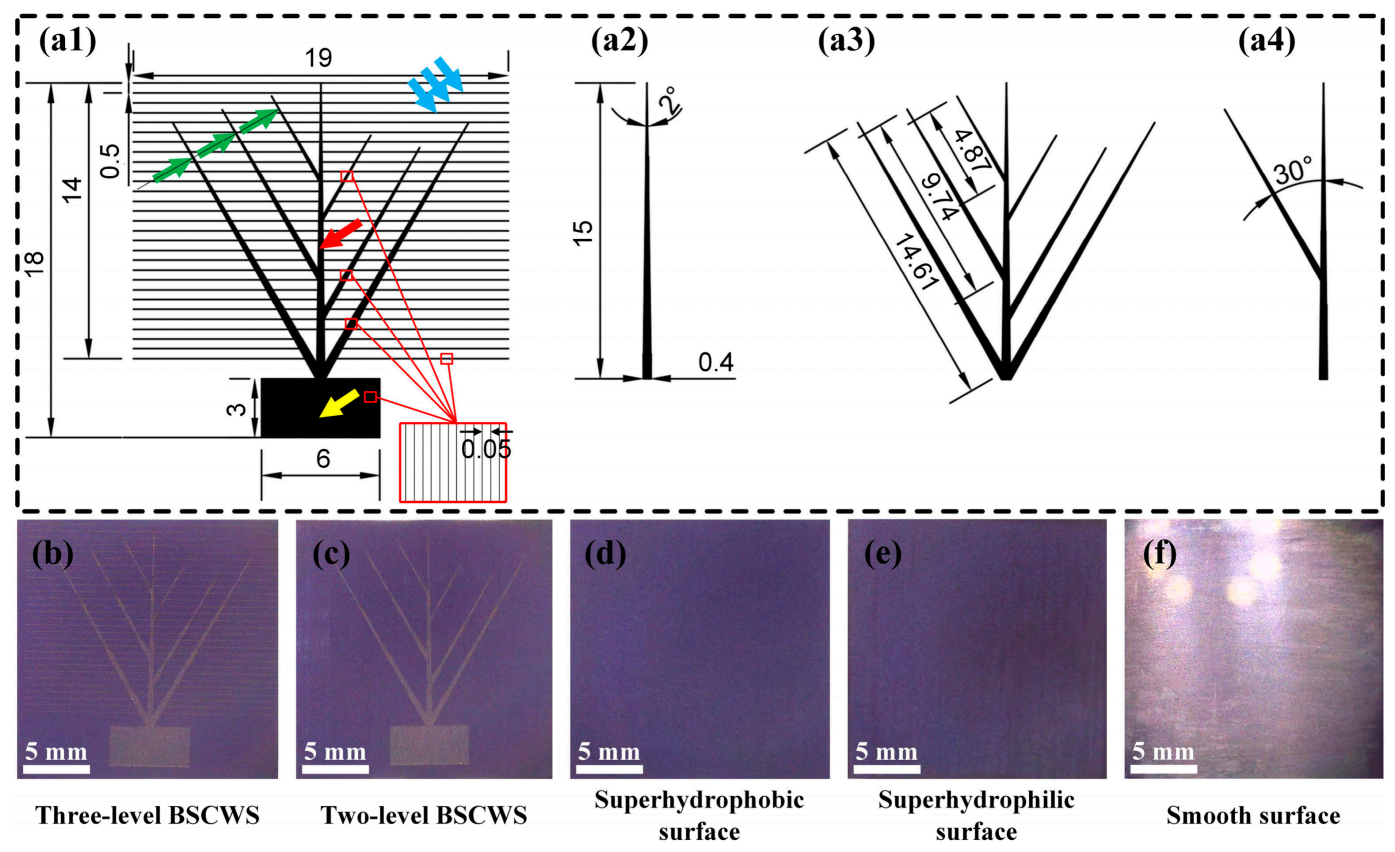
(**a1**–**a4**) Design schematics of the BSCWS (units: mm); (**b**–**f**) photographs of the samples for the three-level BSCWS, two-level BSCWS, superhydrophobic surface, superhydrophilic surface and smooth surface.

The SHBR can prevent droplet adhesion and penetration on the surface [42], while the SHLR can promote rapid droplet nucleation and growth in high-humidity environments [47]. This super-contrast wettability enabled precise control over droplet motion. To enhance water collection efficiency, a novel three-level BSCWS (Figure 5a,b) was designed, integrating large-area water capture, directional transport and centralized storage. This hierarchical structure consisted of (1) primary wedge-shaped channels (red arrows); (2) two-level wedge-shaped channels (green arrows); and (3) three-level rectangular channel arrays (blue arrows). A large rectangular SHLR at the base was used as a “reservoir” (yellow arrows) for water storage. This design aimed to overcome the limitations of traditional wedge-shaped patterned surfaces, which suffered from limited collection areas. The design dimensions of the primary and two-level wedge-shaped channels are shown in Figure 5a1–a4. The apex angle of the wedge-shaped channel was fixed at 2° (Figure 5a2). All the primary and two-level wedge-shaped channels, rectangular channel arrays and the reservoirs featured linear arrays with a spacing of 0.05 mm (Figure 5a1). The included angle between the primary and two-level wedge-shaped channels was uniformly set to 30° (Figure 5a4). Figure 5b–f present physical samples of the three-level BSCWS, the two-level BSCWS (without rectangular channel arrays), the superhydrophobic surface, the superhydrophilic surface and the smooth surface. The last four samples were served as control groups for the subsequent water collection experiments.

#### 3.4.2. Quantitative Analysis of the Water Collection Performance

**Figure 6:**
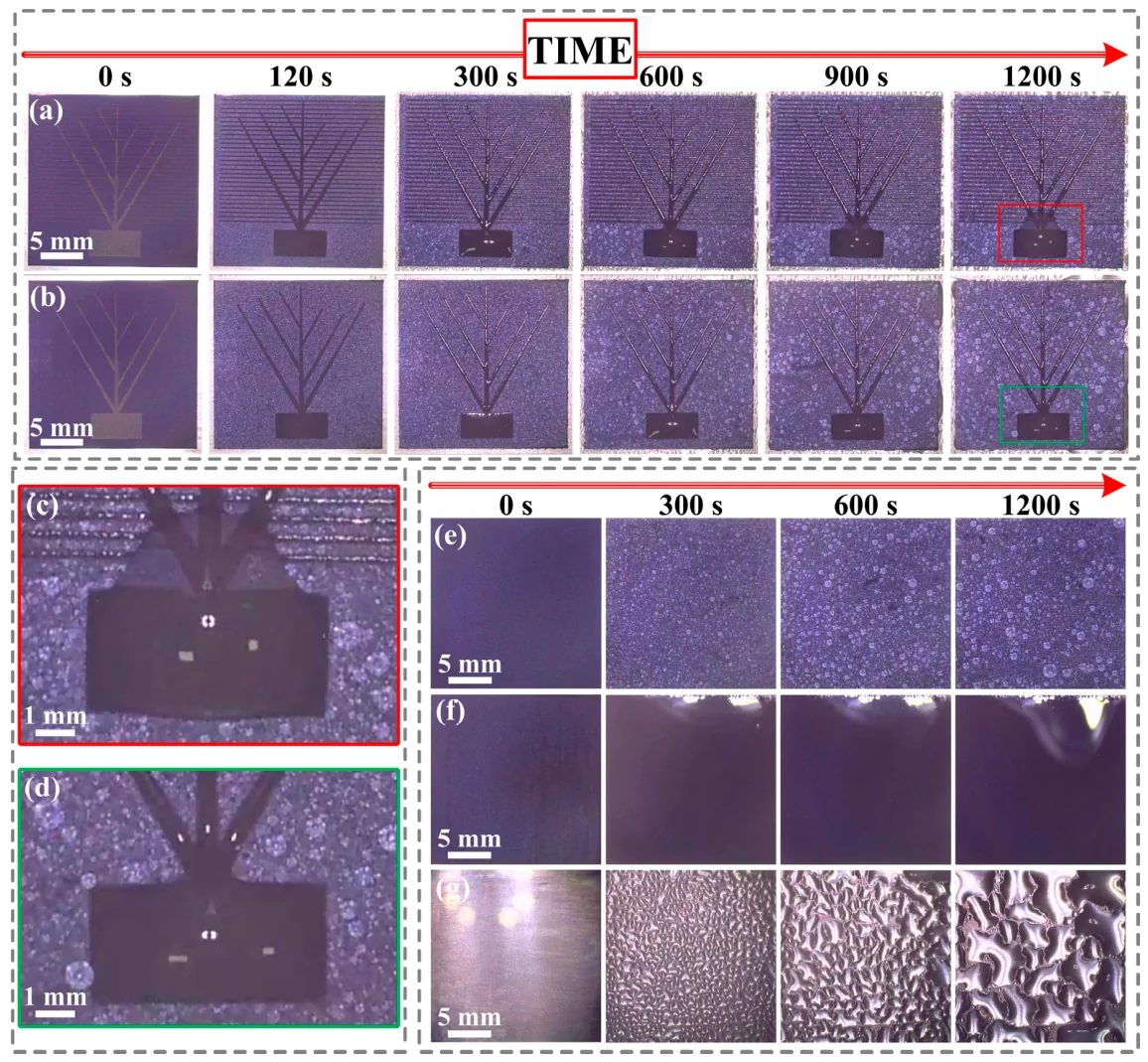
Water condensation processes on five types of surfaces: (**a**) three-level BSCWS; (**b**) two-level BSCWS; (**c**,**d**) enlarged views of the water volume in the reservoirs of the three-level and two-level BSCWSs at 1200 s; (**e**) superhydrophobic surface; (**f**) superhydrophilic surface; (**g**) smooth surface.

The water condensation processes on five surfaces were observed under conditions of 100% relative humidity (RH) and 10 °C. The condensation processes on the three-level and two-level BSCWSs are illustrated in Figure 6a,b. From 0 to 120 s, fog droplets gradually wetted the SHLR. Meanwhile, micro-droplets nucleated and grew on the SHBR, coalescing into larger droplets upon contacting each other. Once these droplets touched the SHLRs, they were instantly absorbed. At 300 s, a continuous water film formed on the SHLR. Driven by the surface energy gradient established at the wettability boundary between the SHBR and SHLR, the water was transported along the rectangular channel arrays, two-level and primary wedge-shaped channels toward the reservoir [27]. At this stage, the water within the rectangular channel arrays, the primary and two-level wedge-shaped channels reached a dynamic equilibrium. From 600 to 1200 s, the continuous accumulation of water in the reservoir exceeded its capacity. This caused a local overflow at the position between the reservoir and the primary channel. The excess water entered the primary wedge-shaped channel and then moved from the base to the apex. This reverse water transport was driven by the effect of surface tension, mainly arising from the strong adsorption of water by the SHLR. In contrast, all the water on the two-level BSCWS remained entirely within the reservoir because less water was collected on the two-level BSCWS within the same period. Figure 6e–g present the condensation processes on the superhydrophobic, superhydrophilic and smooth surfaces. From 0 to 1200 s, the condensed droplets on the superhydrophobic surface maintained a spherical shape, gradually grew and coalesced into larger droplets upon contact. The superhydrophilic surface was completely covered by a continuous water film, whereas the water film on the smooth surface was relatively dispersed due to different wettability from each other. None of these three surfaces could achieve water transport and storage. Although both the three-level and two-level BSCWSs can realize rapid fog water condensation, directional transport and centralized storage, the three-level BSCWS with the rectangular channel arrays achieved a significantly higher water collection capacity than the two-level BSCWS over the same period.

**Figure 7:**
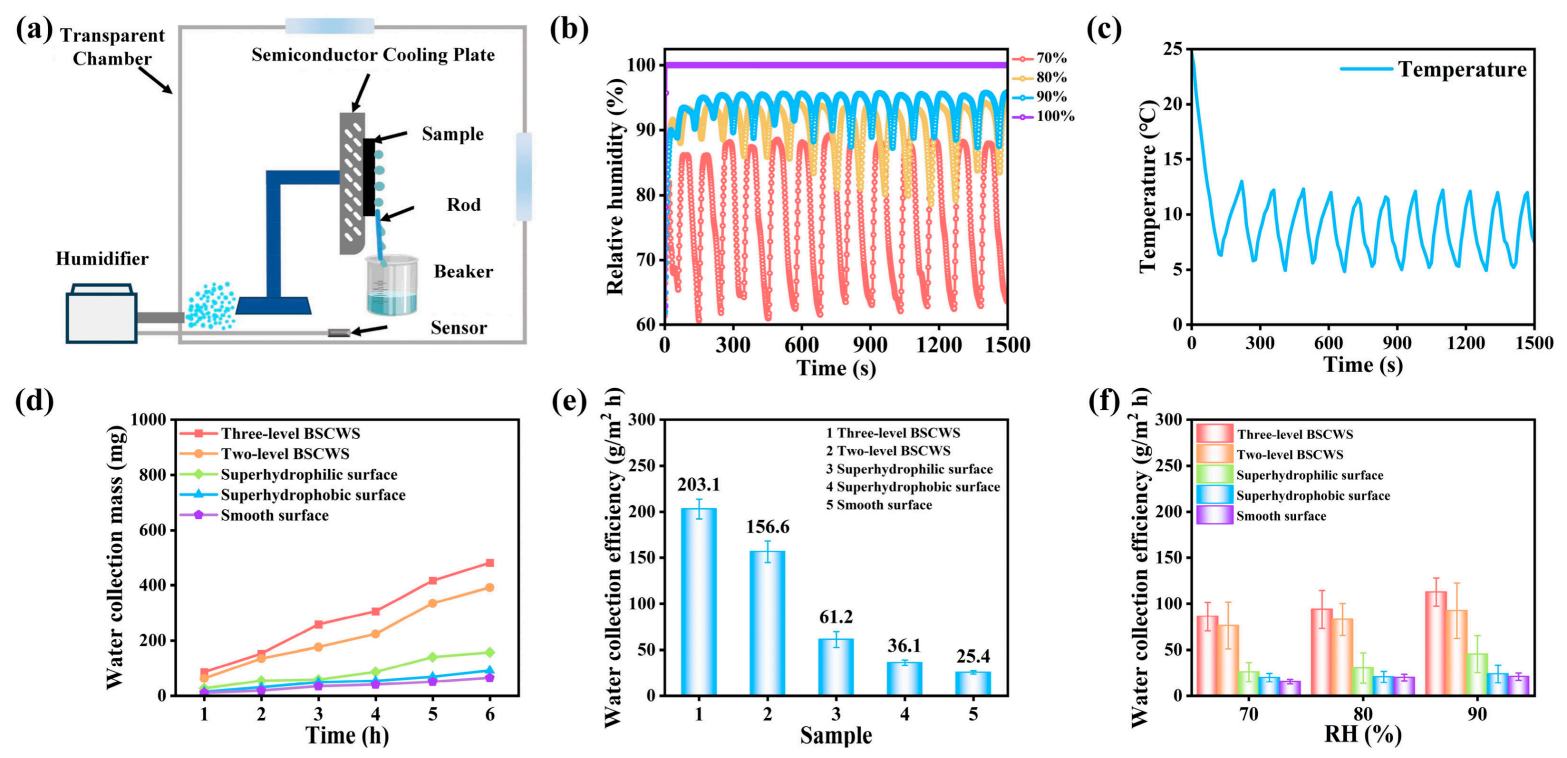
Water collection performance of the five types of surfaces: (**a**) schematic diagram of the custom-built collection device; (**b**) variation curve of the experimental RH; (**c**) variation curve of the surface temperature; (**d**) water collection capacity of the five surfaces within 6 h; (**e**) comparison of the water collection efficiencies among the five surfaces at 100% RH; (**f**) comparison of the water collection efficiencies among the five surfaces at 70%, 80% and 90% RH, respectively.

To quantitatively analyze the water collection performance of the five surfaces, a custom-built experimental setup was employed (Figure 7a). A high-precision ultrasonic humidifier (FX-004, Zhongshan Songbo Co., Ltd., Zhongshan, China) with controllable RH was used as the fog source. The transparent chamber had dimensions of 400 × 150 × 180 cm (L × W × H). The fog source outlet (diameter = 20 mm) was located at the bottom left of the chamber, at horizontal and vertical distances of 20 cm and 15 cm from the sample, respectively. The fog flow rate was 175 mL/h, and the ambient temperature inside the transparent chamber was about 25 ± 2 °C. The fog size was in the range of approximately 0.5–5 μm. The RH inside the transparent chamber was controlled at 70%, 80%, 90% and 100% via a humidity sensor, while the surface temperature was maintained at about 10 °C. The dew point inside the chamber was calculated from the controlled temperature and RH. At 25 °C, the dew points were 25 °C, 23 °C, 21 °C and 19 °C for 100%, 90%, 80% and 70% RH, respectively. The thermoelectric cooler and the samples were vertically fixed on the experimental bracket. A superhydrophilic rod was utilized to rapidly channel the water from the reservoir into the collection container, which eliminated the effect of gravity. The mass of the collected water was measured every 1 h. For each surface, three samples were tested (*n* = 3), and the average values with standard deviations were reported.

The variations in RH and temperature were illustrated in Figure 7b and Figure 7c, respectively. As shown in Figure 7d,e, the collection capacity of five samples increased continuously over time under 100% RH (Figure 7d), which showed an approximately linear relationship with time. After 6 h, the three-level BSCWS achieved the highest water capacity of about 481.7 mg. The capacities for the two-level, superhydrophilic, superhydrophobic and smooth surfaces were 391.3, 156.6, 90.9 and 63.9 mg, respectively. Correspondingly, the three-level BSCWS exhibited the highest collection efficiency of 203.1 ± 10.8 g/m^2^·h, followed by the two-level BSCWS at 156.6 ± 11.7 g/m^2^·h. The collection efficiencies for the superhydrophilic, superhydrophobic and smooth surfaces were 61.2 ± 8.7, 36.1 ± 2.8 and 25.4 ± 1.8 g/m^2^·h, respectively (Figure 7e). Notably, the collection efficiency of the three-level BSCWS surface was 1.3, 3.3, 5.6 and 8.0 times higher than that of the two-level, superhydrophilic, superhydrophobic and smooth surfaces. Furthermore, Figure 7f shows the collection efficiencies of the five surfaces measured at RHs of 70%, 80% and 90%, respectively. It was evident that the collection efficiencies of all five surfaces increased with a rising RH. Under the same RH, the three-level BSCWS consistently maintained the highest water collection efficiency.

#### 3.4.3. Mechanism of Water Collection on the Three-Level BSCWS

**Figure 8:**
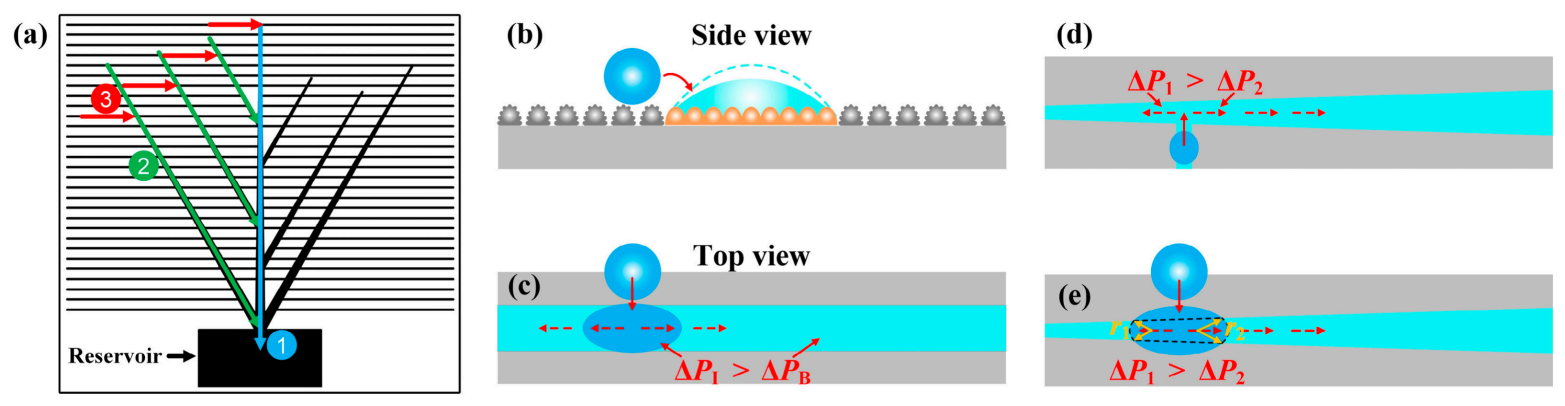
Mechanism analysis for water collection on the three-level BSCWS: (**a**) schematic diagram of the macroscopic mechanism, 1 means water transport from primary wedge-shaped channel to the reservoir, 2 means water transport from two-level to primary wedge-shaped channels, 3 means water transport from rectangular channels to wedge-shaped channels; (**b**,**c**) side and top views of the water collection process in the three-level rectangular channel arrays; (**d**) process of water transport from the three-level channels to the two-level wedge-shaped channels; (**e**) process of condensed droplets entering the primary or two-level wedge-shaped channels.

During the water collection process on the three-level BSCWS, the rectangular channel arrays provide a large area that collects water droplets in the fog and condensed on the SHBR. Benefiting from the excellent anisotropic wettability of the rectangular channel arrays, the captured water rapidly spread along the parallel channels (red arrows in Figure 8a) and is transported to the two-level or primary wedge-shaped channels (green arrows). Subsequently, the water is spontaneously transported via the primary channels into the reservoir (blue arrows). The entire water collection process on the three-level BSCWS is dominated by a dual Laplace pressure-driven mechanism. Firstly, the water collection process in the rectangular channel arrays mainly involves two types of Laplace pressures [11]: balanced Laplace pressure (Δ*P*_B_) and instantaneous Laplace pressure (Δ*P*_I_). As the channels gradually absorb the water from the fog and the droplets condensed on the SHBR, the collected water begins to spread along the rectangular channels. The Δ*P*_B_ is gradually established within the channels and remains equal at any position according to Pascal’s principle. The relationship between Δ*P*_B_ and the water surface curvature slope angle (WSCSA), *β*_B_, in a channel of width *ω* is given by Equation (1):

(1) $$\beta_{B}\left(\omega ,t\right)=\arcsin\left(\frac{\Delta P_{B}\left(t\right)\cdot \omega }{2\gamma }\right)$$

where *γ* is the surface tension of the collected water. This equation indicates that for a given Δ*P*_B_, wider channels possess a larger *β*_B_. In other words, narrow channels hold less water, while wide channels like the reservoir can collect more water. This phenomenon can be observed in Figure 6a,b. When a condensed droplet on the SHBR is drawn into a rectangular channel, a local instantaneous Laplace pressure, Δ*P*_I_, was generated at the position, which is expressed in Equation (2):

(2) $$\Delta P_{I}\left(\omega ,t\right)=\frac{2\gamma \sin\beta_{I}\left(\omega ,t\right)}{\omega }$$

where *β*_I_ is the instantaneous WSCSA. Since *β*_I_ is larger than Δ*β*_B_ at this moment: Δ*P*_I_ can be significantly larger than Δ*P*_B_ (Figure 8b,c) [11]. Consequently, a large local pressure difference (Δ*P*_I_ − Δ*P*_B_) is created. This pressure difference provides a strong driving force that immediately pushes the captured water to other channels until a new equilibrium is reached, which leads to rapid and continuous water transport.

When the water in the three-level rectangular channel arrays or the condensed droplets on the SHBR (Figure 8b,e) enters the primary and two-level channels, the Laplace pressures on both sides of this position on wedge-shaped channels are denoted as Δ*P*_1_ and Δ*P*_2_. An additional Laplace pressure difference arises due to the gradual change in channel width from the apex to the base. The Laplace pressure difference in wedge-shaped primary and two-level channels is expressed as Equation (3):

(3) $$\Delta P=2\gamma \left(\frac{1}{r_{1}}-\frac{1}{r_{2}}\right)=\Delta P_{1}-\Delta P_{2}$$

where *r*_1_ and *r*_2_ are the radius of the curvature at the apex and at the base, thus Δ*P* > 0. The Laplace pressure difference drives the collected water to be transported along the two-level and primary wedge-shaped channels into the reservoir [48,49]. Consequently, the enhanced water collection performance of the large-area capture, directional transport and centralized storage is attributed to the synergistic effect of the dual Laplace pressure difference within the three-level BSCWS.

## 4. Conclusions

In summary, inspired by the anisotropic wettability of indocalamus leaves, the contrast wettability of desert beetle backs and the directional liquid self-transport of cactus spines, we successfully fabricated a hierarchical BSCWS on Ti-6Al-4V alloy by nanosecond laser selective processing and chemical modification. The resulting surface exhibited extreme contrast wettability, with a WCA of 167.4 ± 1.1° in the SHBR and 0° in the SHLR. Chemical analysis revealed that the transition from superhydrophobicity to superhydrophilicity was driven by the thermal decomposition of fluorinated groups and the increase in oxygen-containing groups during the second laser scanning. Furthermore, a novel three-level BSCWS was designed to integrate large-area water capture, directional transport and centralized storage into a single platform. Under 100% RH, the three-level BSCWS achieved a superior water collection efficiency of 203.1 ± 10.8 g/m^2^·h, which was 1.3, 3.3, 5.6 and 8.0 times higher than those of the two-level BSCWS, superhydrophilic, superhydrophobic and smooth surfaces, respectively. The three-level BSCWS also maintained the highest collection efficiencies under 70%, 80% and 90% RH. The excellent water collection performance was attributed to the synergistic effect of the dual Laplace pressure difference within the hierarchical structures, which continuously drove water from the three-level channels through the two-level and primary channels into the reservoir. This work provides a scalable and light-weight strategy for high-efficiency atmospheric water collection in arid environments.

## Figures and Tables

**Table 1:** Laser processing parameters of the BSCWS.

Laser Processing Parameter (Unit)	First Scanning	Second Scanning
Frequency (kHz)	20	
Pulse width (ns)	100	
Laser beam wavelength (nm)	1064	
Laser spot diameter (μm)	50	
Power (W)	3	3
Scanning interval (μm)	3	50
Scanning speed (mm/s)	200	100
Number of scans (times)	2	2
Processing time (s)	1333.2	14.8
Processing efficiency (s/100 mm^2^)	333.3	3.7

**Table 2:** Surface roughness of the BSCWS, SHLR and SHBR.

Surface	Sa (μm)	Sz (μm)	Sp (μm)	Sv (μm)	Sq (μm)
BSCWS	8.50 ± 0.15	139.43 ± 9.91	56.50 ± 4.06	82.93 ± 8.49	10.66 ± 0.20
SHBR	5.38 ± 0.38	64.40 ± 12.52	25.04 ± 6.77	39.35 ± 5.91	6.69 ± 0.41
SHLR	9.55 ± 0.29	139.67 ± 9.11	55.89 ± 7.13	83.77 ± 4.55	11.68 ± 0.35

**Table 3:** Elemental contents of the BSCWS, SHLR and SHBR (atomic ratio %).

Element	BSCWS	SHBR	SHLR
C	6.77	10.61	4.10
O	47.37	43.16	50.47
Ti	29.96	20.36	40.60
Al	2.36	1.70	2.99
V	1.39	0.99	1.83
F	11.88	22.58	0
Si	0.28	0.61	0

**Table 4:** Relative contents of C1s functional groups in the SHLR and SHBR (%).

Functional Group	SHBR	SHLR
C−C/C−H	50.31	64.02
C−O	12.70	33.55
C=O	7.90	2.43
−CF_2_	24.92	0
−CF_3_	4.16	0
C−O and C=O	20.60	35.98

## Data Availability

Data are available upon reasonable request.

## Supplementary Materials

The following supporting information can be downloaded at: https://www.mdpi.com/article/10.3390/biomimetics11100759/s1, Figure S1: (a) Adhesion force of the SHBR; (b) sliding angles measured in the perpendicular and parallel directions on the BSCWS.
