# Supplementary material for: Fabrication of a Hierarchical Bioinspired Super-Contrast Wettability Surface for Enhanced Atmospheric Water Collection
Source: Biomimetics (Basel). 2026 Oct 20;11(10):759. doi: 10.3390/biomimetics11100759 (PMC13702612; doi:10.3390/biomimetics11100759)
Supplement: Supplementary file 1 [file biomimetics-11-00759-s001.zip › biomimetics-4578804-supplementary.pdf]

# Supplementary material

# Fabrication of a Hierarchical Bioinspired Super-Contrast Wettability Surface for Enhanced Atmospheric Water Collection

**Ming Li [1], Bo Wang [1,*], Bingyang Wang [2], Yanling Wan [3]**

1 School of Intelligent Manufacturing and Electrical Engineering, Guangzhou Institute of Science and Technology, Guangzhou 510540, China; lmingdr@163.com (M.L.); wanborn@163.com (B.W.)
2 College of Mechanical and Automotive Engineering, Ningbo University of Technology, Ningbo 315211, China; bywang2024@nbut.edu.cn (B.Y.-W.)
3 Ministry of Education Key Laboratory of Cross-scale Micro and Nano Manufacturing, Changchun University of Science and Technology, Changchun 130022, China; wanly@cust.edu.cn (Y.L.-W)
* Correspondence: wanborn@163.com (B.W.)

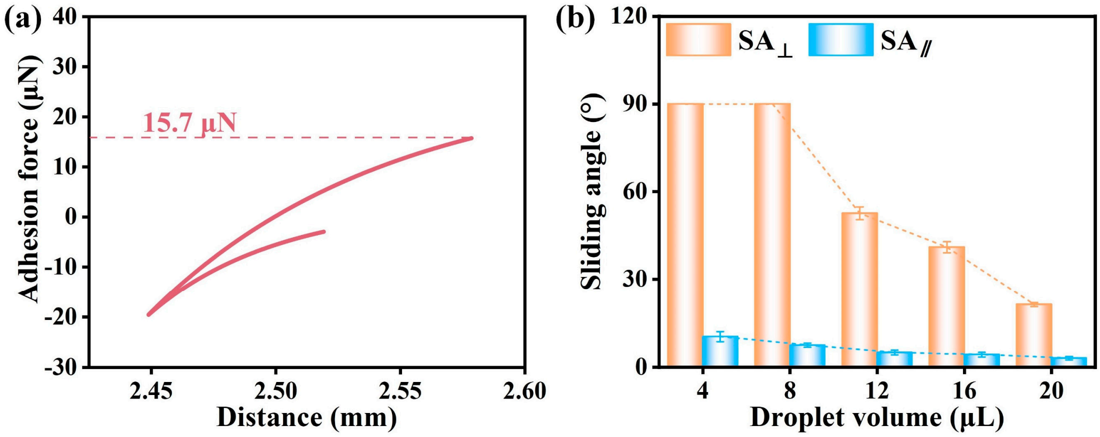


**Figure S1. (a) Adhesion force of SHBR; (b) Sliding angles measured in perpendicular and parallel directions on BSCWS.**
